# 

**Published:** 1894-10-20

**Authors:** 


**Notices of Births, Deaths, and Marriages** are inserted in this column at a charge of 2s. 6d. for an announcement not exceeding four lines.

**Notice to Advertisers and Subscribers.**

All Advertisements, Orders for Copies of The Hospital, and Business Communications should be addressed to **The Publisher, NOT TO THE EDITOR,** The Hospital, 428, Strand, W.C.

The Cost of Subscription, post free, is as follows:—Per week, 2½d.; per quarter, 2s. 8d.; per half-year, 5s. 3d.; per annum, 10s. 6d. Foreign:—Per Annum, 12s. 8d.; payable, in all cases, in advance.

### NOTICE TO CORRESPONDENTS.

All MS., Letters, Books for Review, and other matters intended for the Editor should be addressed The Editor, The Lodge, Porchester Square, London, W.

The Editor cannot undertake to return rejected MS., even when accompanied by stamped directed envelope.

**"Burdett's Hospital Annual," 1894.**

Fifth year of publication. 900 pp. Boards, gilt lettering. A most complete guide to British, Colonial, Indian, and American Hospitals, Dispensaries, Nursing and Convalescent Institutions, and Asylums. Price 5s. Published by The Scientific Press (Limited), 428, Strand, W.C.

**NOTICE.—The Index for Vol. XV. (ending March 31st, 1894) is on sale at the Office of this Journal, price 2½d., post free.**

### SCRAPS AND GLEANINGS.

The amount produced by the Hospital Tuesday appeal for the Ipswich Hospital was £180 this year.

Hospital Sunday at Cork realised about £400, which sum is a great improvement on last year's collection.

The French Association for the Advancement of Science will assemble next year at Caen, from August 9th to 15th.

There are no fewer than 22 general and 16 special hospitals in Chicago, and an accommodation is supplied of 3,409 beds.

Towards the credit of the proposed Eye, Ear, and Throat Hospital, at Londonderry, the sum of £877 has already been lodged in the bank.

A sum of £500 has been raised at Swindon towards defraying the expenses of the new wing in course of erection to the Victoria Hospital.

A legacy of £1,000, free of duty, has been bequeathed to the North Riding Infirmary, Middlesburgh, by Mr. Robert Hymers, of Stokestey.

The Accrington Cottage Hospital Fund now amounts to nearly £1,400, and this sum has been collected within a week of the appeal being issued.

The collection which was made in the streets of Ipswich on Hospital Saturday has added considerably to the funds of the East Suffolk Hospital.

Plans for the erection of the Kincardine Hospital have been received, but the decision on these is held over until the question of site has been finally agreed upon.

Last year no fewer than 160 candidates applied to the International Medical Nursing Institute, New York, to be received to prepare for the medical nursing service.

In aid of the German Hospital in Dalston, the German Company at the Opera Comique, under the direction of Mr. Charles Maurice, gave a special performance on Saturday afternoon.

The scheme for an annual Hospital Saturday Collection is being promulgated at Ashton-under-Lyne, with a view to benefiting the local Infirmary and Children's Hospital.

Tenders for building the new fever wing in extension of the Meath Hospital and County Dublin Infirmary have been prepared, and the accommodation will provide for forty patients.

The Cottage Hospital, Watford, is in much need of funds, and having a deficit due to the treasurer the Hospital Saturday Committee decided to allot £65 towards defraying the existing debt.

It has for some time been considered necessary to extend the premises of the North-Western Hospital, Haverstock Hill. Plans for this purpose have now been approved of by the Metropolitan Asylums Board.

The new infirmary for the sick poor of the Lewisham Union has been opened at High-street, Lewisham. The new building will provide for as many as 372 patients, and the cost is little under £73,000.

Hospital Saturday at Gravesend appears really to be little else than a farce. It seems that the annual collection this year realised £2 3s. 9d., fourteen boxes being in the streets for twelve hours resulted in the above small sum.

The Viceroy Li Hung Chang has established at Tientsin an Imperial Medical College. A four years' graduated course is here required, and in connection with the institute a well-equipped hospital has been added.

The Duc d'Orleans has sent a thousand francs to M. Magnard, of the *Figaro*, as a subscription towards the establishment of what is termed systematic vaccination for croup, or in other words the serum treatment for diphtheria.

An average deficit of £115 per annum on the accounts of the Lowestoft Hospital during the last four years shows that internal vigilance in financial matters is the price which must be paid if the safety of the hospital pecuniarily is to be permanently assured.

The Rev. John Henn has announced his intention to withdraw from the office of secretary to the Manchester Sunday and Saturday collection. The amount raised during the quarter of a century Mr. Henn has presided officially has not been far short of £190,000.

Great efforts have been made during the last twelve months to place the finances of Addenbrooke's Hospital at Cambridge on a better footing. Large as was the amount contributed last year, it falls about £200 short of the necessary expenditure, which is estimated from £6,335 to £6,355 for the twelve months.

The financial position of the Devonshire Hospital and Buxton Bath Charity continues to be generally satisfactory. An increase in casual subscriptions is noticeable in the last report. A sale of work was instituted lately on behalf of the hospital, from which an additional sum of money was obtained.

A grand bazaar was held last week in aid of the building fund of the Fleetwood Cottage Hospital. A substantial amount was realized by the sale of articles and "money making" side shows. Close upon £1,000 is yet required to complete the necessary sum for the building and furnishing of the new infirmary.

A ward in the newly executed extension of the Wolverhampton Woman's Hospital has been furnished by Mrs. Appleby, of Wolverhampton. This will be called the "Broadbent," in memory of the benefactress's mother. Donations are urgently needed to meet increased expenses of this very deserving charity.

An interesting competition was recently entered into at the instance of a scientific contemporary who took the greatest number of votes on which was the most popular beef essence in the market. Eleven thousand five hundred and sixty-three coupons were sent in, out of which 4,318 gave the foremost place to "Bovril."

The Eye and Ear Hospital at Southampton held last week their annual "Pound Day," when the whole of the day gifts were brought in pounds to the institution, which does a large amount of good among the poorer inhabitants of the town. It is proposed to extend the ever-increasing sphere of this hospital, and some valuable property has been acquired for this purpose. Two gifts of £1,000 each have been received in view of this outlay from Mr. A. Barlow and Mr. W. Garton.

### The Student of Medicine.

### VACANCIES.

### Resident Medical Officers.

Carnarvonshire and Anglesey Infirmary and Dispensary, Bangor—House Surgeon. Must be registered to practise in Medicine and Surgery, and have a knowledge of the Welsh language. Salary £70 per annum, with board and lodging. Applications to the Secretary, before October 27th.

Great Northern Central Hospital, Holloway Road, N.—House Physician. Salary £60 per annum with board, lodging, and washing. Applications to Lewis H. Glenton Kerr, Secretary, by October 23rd.

London Lock Hospital, Harrow Road, W.—House Surgeon. Salary £50 per annum, with board, lodging, and washing. Applications to the Secretary before October 23rd.

Halifax Infirmary and Dispensary.—House Surgeon. Unmarried. Salary £80 per annum, advancing £10 per annum up to £100, with residence, board, and washing. Applications to Oates Webster, Secretary, by October 24th.

South Devon and East Cornwall Hospital, Plymouth.—Assistant House Surgeon. No salary, board and residence provided. Applications and testimonials to J. Walter Wilson, Honorary Secretary, before October 20th.

Stourbridge Dispensary.—House Surgeon and Secretary. Must have a qualification in Medicine and Surgery from one of the Colleges of England, Scotland, or Ireland. Salary £120 per annum, with furnished rooms, coals, and gas, with an allowance of £25 for travelling expenses. Applications to T. F. Bland, Honorary Secretary, Norton, Stourbridge, before October 24th.

### THE STUDENT OF MEDICINE—*continued.*

St. Pancras and Northern Dispensary, 126, Euston Road, N.W.—Resident Medical Officer, doubly qualified. Salary £105 per annum, with residence and attendance; and a Physician Accoucheur. Applications to H. Peter Bodkin, Honorary Secretary, 23, Gordon Street, Gordon Square, by October 22nd.

#### Non-Resident Medical Officers.

North London Hospital for Consumption, Hampstead.—Assistant Physician. Candidates must be graduated in Medicine of a University of the United Kingdom or Fellows or Members of the Royal College of Physicians of London. Applications to L. F. Hill, M.A., Secretary, at the Office, before November 3rd.

Hospital for Sick Children, Great Ormond Street, W.C.—Surgical Registrar and Anæsthetist. Appointment for one year, an honorarium of £40 being voted at the expiration of that term. Candidates must appear before the Joint Committee on October 24th.

Mason College, Birmingham.—Professorship of Midwifery. Applications to G. H. Morley, Secretary, before October 27th.

The Royal Hospital for Diseases of the Chest, City Road, E.C.—Assistant Physician. Candidates must be Fellows or Members of the Royal College of Physicians of London. Applications to J. Harrold, Secretary, before October 24th.

University College, London.—Surgical Registrar. Applications to J. M. Horsburgh, M.A., Secretary, by October 22nd.

